# Genome sequence of the *Thermotoga thermarum* type strain (LA3^T^) from an African solfataric spring

**DOI:** 10.4056/sigs.3016383

**Published:** 2014-05-15

**Authors:** Markus Göker, Stefan Spring, Carmen Scheuner, Iain Anderson, Ahmet Zeytun, Matt Nolan, Susan Lucas, Hope Tice, Tijana Glavina Del Rio, Jan-Fang Cheng, Cliff Han, Roxanne Tapia, Lynne A. Goodwin, Sam Pitluck, Konstantinos Liolios, Konstantinos Mavromatis, Ioanna Pagani, Natalia Ivanova, Natalia Mikhailova, Amrita Pati, Amy Chen, Krishna Palaniappan, Miriam Land, Loren Hauser, Yun-juan Chang, Cynthia D. Jeffries, Manfred Rohde, John C. Detter, Tanja Woyke, James Bristow, Jonathan A. Eisen, Victor Markowitz, Philip Hugenholtz, Nikos C. Kyrpides, Hans-Peter Klenk, Alla Lapidus

**Affiliations:** 1 Leibniz Institute DSMZ - German Collection of Microorganisms and Cell Cultures, Braunschweig, Germany; 2 DOE Joint Genome Institute, Walnut Creek, California, USA; 3 Los Alamos National Laboratory, Bioscience Division, Los Alamos, New Mexico, USA; 4 Biological Data Management and Technology Center, Lawrence Berkeley National Laboratory, Berkeley, California, USA; 5 Oak Ridge National Laboratory, Oak Ridge, Tennessee, USA; 6 HZI – Helmholtz Centre for Infection Research, Braunschweig, Germany; 7 University of California Davis Genome Center, Davis, California, USA; 8 Australian Centre for Ecogenomics, School of Chemistry and Molecular Biosciences, The University of Queensland, Brisbane, Australia; 9 Department of Biological Sciences, King Abdulaziz University, Jeddah, Saudi Arabia; 10 Theodosius Dobzhansky Center for Genome Bionformatics, St. Petersburg State University, St. Petersburg, Russia; 11 Algorithmic Biology Lab, St. Petersburg Academic University, St. Petersburg, Russia

**Keywords:** anaerobic, motile, thermophilic, chemoorganotrophic, solfataric spring, outer sheath-like structure, *Thermotogaceae*, GEBA

## Abstract

*Thermotoga thermarum* Windberger et al. 1989 is a member to the genomically well characterized genus *Thermotoga* in the phylum ‘*Thermotogae*’. *T. thermarum* is of interest for its origin from a continental solfataric spring vs. predominantly marine oil reservoirs of other members of the genus. The genome of strain LA3T also provides fresh data for the phylogenomic positioning of the (hyper-)thermophilic bacteria. *T. thermarum* strain LA3^T^ is the fourth sequenced genome of a type strain from the genus *Thermotoga*, and the sixth in the family *Thermotogaceae* to be formally described in a publication. Phylogenetic analyses do not reveal significant discrepancies between the current classification of the group, 16S rRNA gene data and whole-genome sequences. Nevertheless, *T. thermarum* significantly differs from other *Thermotoga* species regarding its iron-sulfur cluster synthesis, as it contains only a minimal set of the necessary proteins. Here we describe the features of this organism, together with the complete genome sequence and annotation. The 2,039,943 bp long chromosome with its 2,015 protein-coding and 51 RNA genes is a part of the Genomic Encyclopedia of Bacteria and Archaea project.

## Introduction

Strain LA3^T^ (= DSM 5069 = NBRC 107925) is the type strain of the species *Thermotoga thermarum* [1], one out of currently nine species in the genus *Thermotoga* [2]. The genus name was derived from the Greek word thermê, heat, and the Latin word toga, Roman outer garment; *Thermotoga*, the hot outer garment [3]; the species epithet was derived from the Latin word thermarum, of warm springs, of warm baths [1]. Strain LA3^T^ was originally isolated from a hot continental solfataric spring in Lac Abbé, southwest of Asbalto, Djibouti [1]. Here we present a summary classification and a set of features for *T. thermarum* LA3^T^, together with the description of the genomic sequencing and annotation. 

## Features of the organism

### 16S rRNA gene analysis

The single genomic 16S rRNA gene sequence of *T. thermarum* LA3^T^ was compared with the Greengenes database [4] for determining the weighted relative frequencies of taxa and (truncated [5]) keywords as previously described [6,7]. The most frequently occurring genera were *Thermotoga* (53.9%), *Thermosipho* (29.1%), *Fervidobacterium* (11.0%), *Caldicellulosiruptor* (2.5%) and *‘Thermopallium*' (1.4%) (130 hits in total). Regarding the two hits to sequences from members of the species, the average identity within HSPs was 100.0%, whereas the average coverage by HSPs was 95.7%. Regarding the 37 hits to sequences from other members of the genus, the average identity within HSPs was 92.1%, whereas the average coverage by HSPs was 98.4%. Among all other species, the one yielding the highest score was *Thermotoga hypogea* (U89768), which corresponded to an identity of 94.2% and an HSP coverage of 99.1%. (Note that the Greengenes database uses the INSDC (= EMBL/NCBI/DDBJ) annotation, which is not an authoritative source for nomenclature or classification.) The highest-scoring environmental sequence was DQ675048 ('microbial production water -temperature petroleum reservoir clone QHO-B59'), which showed an identity of 99.0% and an HSP coverage of 82.0%. The most frequently occurring keywords within the labels of all environmental samples which yielded hits were 'microbi' (5.6%), 'temperatur' (3.2%), 'spring' (3.0%), 'hot' (2.6%) and 'thermophil' (2.3%) (117 hits in total). The most frequently occurring keywords within the labels of those environmental samples which yielded hits of a higher score than the highest scoring species were 'microbi, petroleum, reservoir, temperatur' (11.8%), 'product, water' (6.0%) and 'aggregate-form, biodegrad, crude-oil-adh, fluid, niiboli, oilfield, produc' (5.8%) (2 hits in total). Some of these keywords fit well to the known ecology of *T. thermarum*.

**Figure 1:**
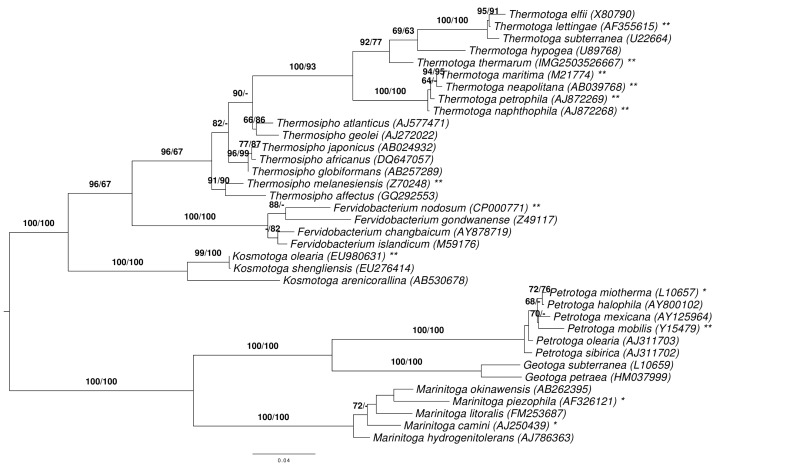
shows the phylogenetic neighborhood of *T. thermarum* in a 16S rRNA gene based tree. The sequence of the single 16S rRNA gene copy in the genome does not differ from the previously published 16S rRNA gene sequence (AB039769).

Figure 1. Phylogenetic tree highlighting the position of *T. thermarum* relative to the type strains of the other species within the family *Thermotogaceae*. The tree was inferred from 1,373 aligned characters [8,9] of the 16S rRNA gene sequence under the maximum likelihood (ML) criterion [10] and rooted [11] as previously described [7]. The branches are scaled in terms of the expected number of substitutions per site. Numbers adjacent to the branches are support values from 250 ML bootstrap replicates [12] (left) and from 1,000 maximum-parsimony bootstrap replicates [13] (right) if larger than 60%. Lineages with type strain genome sequencing projects registered in GOLD [14] are labeled with one asterisk, those also listed as 'Complete and Published' with two asterisks [15-17] (for *T. neapolitana* and *T. naphthophilia*e see CP000916 and CP001839, respectively, and for *Petrotoga mobilis* CP000879).

The tree depicted in Figure 1 reveals discrepancies between the current classification of the group and 16S rRNA phylogenetic analysis. First, *Thermotoga* is nested within a paraphyletic *Thermosipho*, but without support under the maximum-parsimony criterion. Second, when drafting this study *Thermococcoides shengliensis* had not yet been assigned to *Kosmotoga* as *K. shengliensis* and thus was nested within paraphyletic *Kosmotoga* with almost maximal to maximal support (99-100%). To assess whether the disagreement between the 16S rRNA data and the classification regarding *Thermosipho* and *Thermotoga* was statistically significant, we conducted constraint-based paired-site tests as described earlier [18], using the assignment of the species to genera as depicted in Figure 1 (assigning *T. shengliensis* to *Kosmotoga*) as constraint. Search under the maximum-likelihood criterion yielded a best tree with a score of -9,500.82 if the search was unconstrained but a tree with a log likelihood of -9,521.15 under the constraint; this was not significantly worse in the SH test as implemented in RAxML (α = 0.05). Hence, the *Thermosipho*-*Thermotoga* problem seems to be negligible.

In contrast, the only recently fixed *Kosmotoga-Thermococcoides* problem was much more apparent in the 16S rRNA gene data. It is also of distinct origin, as it seems to be caused by confusing treatments of issues of nomenclature. In 2009, DiPippo and coworkers [19] described *Kosmotoga olearia* as novel species in a novel genus. In the following year, Feng and colleagues [20], without comparing their newly isolated strain to the type strain of *K. olearia* (which might not yet have been publicly available when the study presented in [20] was conducted), published *T. shengliensis*, also in a novel genus. More recently, Nunoura et al. [21] added *K. arenicorallina* to the genus *Kosmotoga*. These authors also realized that *T. shengliensis* and *K. olearia* are more closely related to each other than *K. arenicorallina* to *K. olearia* and thus suggested to place *T. shengliensis* in *Kosmotoga* as *K. shengliensis* because *Kosmotoga* has priority over Thermococcoides.

Whereas the validation of *K. arenicorallina* was accepted by the International Journal of Systematic and Evolutionary Microbiology (IJSEM) [22], *K. shengliensis* was at first not accepted by the editors of IJSEM with reference to rule 31a [2] of the Bacteriological Code (Nunoura, pers. comm.). Probably the editors opined that a DNA-DNA hybridization experiment [23] between the type strains of *K. olearia* and *T. shengliensis* should be conducted to assess whether both represent a single or two distinct species. In the meantime, the name *K. shengliensis* has been validated, however. The advantages of this solution can be demonstrated by considering the number of conflicts between data and classification. With *Thermococcoides shengliensis* in use, the classification of the group caused one obvious problem, the paraphyly of *Kosmotoga* (Fig. 1), and one potential problem, that *K. shengliensis* and *T. shengliensis* might be conspecific. By accepting the proposal in [21] to assign *T. shengliensis* to the genus *Kosmotoga*, the first problem was solved and the second problem was not worsened.

**Figure 2:**
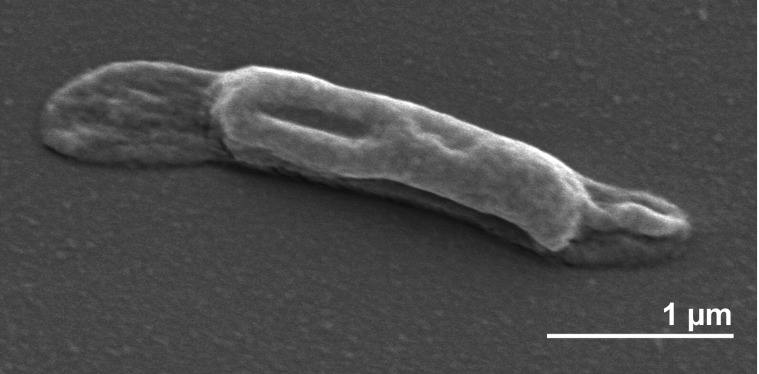
Scanning electron micrograph of *T. thermarum* LA3^T^

*T. thermarum* LA3^T^ is Gram-negative-staining and rod-shaped, with a sheath that extends past the ends of the cell (Figure 2). Cells were reported to be 0.6 μm in width and 1.5-11 μm in length [1]. Flagella and motility were observed [1] (Table 1). Growth occurred between 55°C and 84°C with an optimum at 70°C [1]. The pH range for growth was 5.5-9.0 with 7.0 as the optimum [1]. The salinity range for growth was 0.2% to 0.55% NaCl with 0.35% as the optimum value [1]. Yeast extract was required for growth, and addition of glucose, maltose, or starch significantly increased cell yield [1]. H_2_ and S^0^ both inhibited growth, and H_2_S was not formed from S^0^ [1].

**Table 1:** Classification and general features of *T. thermarum* LA3^T^ according to the the MIGS recommendations [24].

MIGS ID	Property	Term	Evidence code
	Current classification	Domain *Bacteria*	TAS [25]
		Phylum ‘*Thermotogae*’	TAS [26,27]
		Class *Thermotogae*	TAS [26,28]
		Order *Thermotogales*	TAS [26,29]
		Family *Thermotogaceae*	TAS [26,30]
		Genus *Thermotoga*	TAS [3,31]
		Species *Thermotoga thermarum*	TAS [1,32]
		Type strain LA3	TAS [1]
	Gram stain	negative	TAS [1]
	Cell shape	rods with a ‘toga’ (a sheath-like structure)	TAS [1]
	Motility	motile	TAS [1]
	Sporulation	not reported	
	Temperature range	thermophile, 55-84°C	TAS [1]
	Optimum temperature	70°C	TAS [1]
	Salinity	0.2 - 0.6% NaCl (w/v), opt 0.35%	TAS [31]
MIGS-22	Oxygen requirement	anaerobe	TAS [1]
	Carbon source	yeast extract, glucose, maltose, starch	TAS [1]
	Energy metabolism	chemoorganotroph	NAS
MIGS-6	Habitat	low salinity hydrothermal well water	TAS [1]
MIGS-15	Biotic relationship	free living	TAS [1]
MIGS-14	Pathogenicity	none	NAS
	Biosafety level	1	TAS [33]
MIGS-23.1	Isolation	continental solfataric spring	TAS [1]
MIGS-4	Geographic location	Lac Abbé, southwest of Asbalto, Djibouti	TAS [1]
MIGS-5	Sample collection time	1989 or earlier	NAS
MIGS-4.1	Latitude	11.162	NAS
MIGS-4.2	Longitude	41.781	NAS
MIGS-4.3	Depth	not reported	
MIGS-4.4	Altitude	5 – 30 m	TAS [1]

Evidence codes - TAS: Traceable Author Statement (i.e., a direct report exists in the literature); NAS: Non-traceable Author Statement (i.e., not directly observed for the living, isolated sample, but based on a generally accepted property for the species, or anecdotal evidence). Evidence codes are from of the Gene Ontology project [34].

### Chemotaxonomy

The analysis of complex lipids in strain LA3^T^ showed that they were similar to those of *T. maritima* except that the less polar glycolipid was absent [1]. Analysis of core lipids showed that strain LA3^T^ had one unidentified core lipid that was not present in *T. maritima* [1].

## Genome sequencing and annotation

### Genome project history

This organism was selected for sequencing on the basis of its phylogenetic position [35], and is part of the Genomic Encyclopedia of *Bacteria* and *Archaea* project [36,37]. The genome project is deposited in the Genomes On Line Database [14] and the complete genome sequence is deposited in GenBank. Sequencing, finishing and annotation were performed by the DOE Joint Genome Institute (JGI). A summary of the project information is shown in Table 2.

**Table 2:** Genome sequencing project information

MIGS ID	Property	Term
MIGS-31	Finishing quality	Finished
MIGS-28	Libraries used	Three genomic libraries: one 454 pyrosequence standard librariy, one 454 PE library (10 kb insert size), one Illumina library
MIGS-29	Sequencing platforms	Illumina GAii, 454 GS FLX Titanium
MIGS-31.2	Sequencing coverage	142.2 × Illumina; 6.8 × pyrosequence
MIGS-30	Assemblers	Newbler version 2.3-PreRelease-10/20/2009, Velvet, phrap version SPS - 4.24
MIGS-32	Gene calling method	Prodigal
	INSDC ID	CP002351
	GenBank Date of Release	November 21, 2011
	GOLD ID	Gc01826
	NCBI project ID	41517
	Database: IMG	2503508007
MIGS-13	Source material identifier	DSM 5069
	Project relevance	Tree of Life, GEBA

### Growth conditions and DNA isolation

*T. thermarum* strain LA3^T^, DSM 5069, was grown anaerobically in DSMZ medium 498 (*Thermotoga* II medium) [38] at 80°C. DNA was isolated from 0.5-1 g of cell paste using MasterPure Gram-positive DNA purification kit (Epicentre MGP04100) following the standard protocol as recommended by the manufacturer with modification st/DL for cell lysis as described in Wu *et al.* 2009 [37]. DNA is available through the DNA Bank Network [39].

### Genome sequencing and assembly

The genome was sequenced using a combination of Illumina and 454 sequencing platforms. All general aspects of library construction and sequencing can be found at the JGI website [40]. Pyrosequencing reads were assembled using the Newbler assembler (Roche). The initial Newbler assembly, consisting of one contig in one scaffold, was converted into a phrap [41] assembly by making fake reads from the consensus, to collect the read pairs in the 454 paired end library. Illumina GAii sequencing data (290.0 Mb) was assembled with Velvet [42] and the consensus sequences were shredded into 1.5 kb overlapped fake reads and assembled together with the 454 data. The 454 draft assembly was based on 14.0 Mb 454 draft data and all of the 454 paired end data. Newbler parameters are -consed -a 50 -l 350 -g -m -ml 20. The Phred/Phrap/Consed software package [41] was used for sequence assembly and quality assessment in the subsequent finishing process. After the shotgun stage, reads were assembled with parallel phrap (High Performance Software, LLC). Possible mis-assemblies were corrected with gapResolution [40], Dupfinisher [43], or sequencing cloned bridging PCR fragments with subcloning. Gaps between contigs were closed by editing in Consed, by PCR and by Bubble PCR primer walks (J.-F. Chang, unpublished). A total of 16 additional reactions were necessary to close gaps and to raise the quality of the finished sequence. Illumina reads were also used to correct potential base errors and increase consensus quality using a software Polisher developed at JGI [44]. The error rate of the completed genome sequence is less than 1 in 100,000. Together, the combination of the Illumina and 454 sequencing platforms provided 149.0 × coverage of the genome. The final assembly contained 414,118 pyrosequence and 1,166,274 Illumina reads.

### Genome annotation

Genes were identified using Prodigal [45] as part of the Oak Ridge National Laboratory genome annotation pipeline, followed by a round of manual curation using the JGI GenePRIMP pipeline [46]. The predicted CDSs were translated and used to search the National Center for Biotechnology Information (NCBI) non-redundant database, UniProt, TIGRFam, Pfam, PRIAM, KEGG, COG, and InterPro databases. These data sources were combined to assert a product description for each predicted protein. Additional gene prediction analysis and functional annotation was performed within the Integrated Microbial Genomes - Expert Review (IMG-ER) platform [47].

## Genome properties

**Figure 3:**
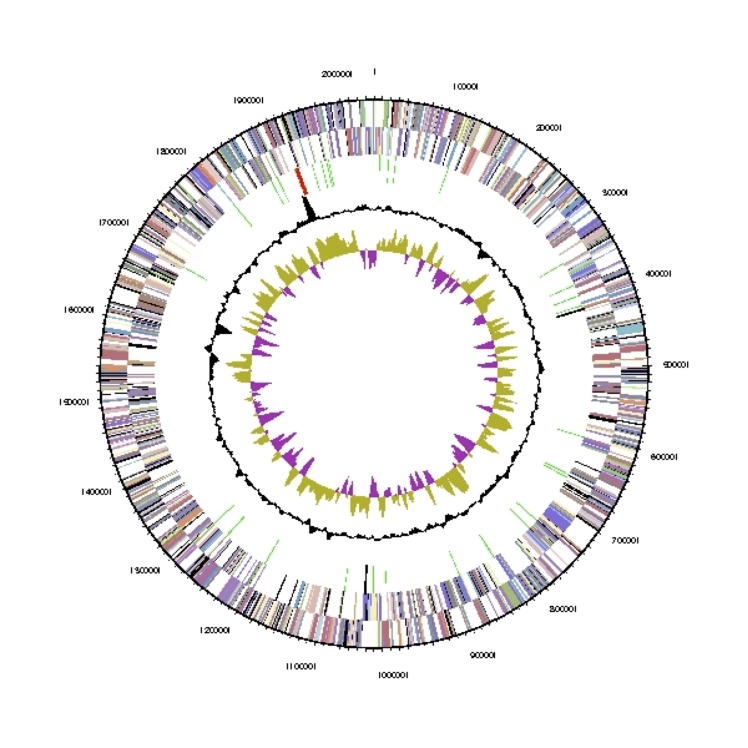
Graphical map of the chromosome. From outside to the center: Genes on forward strand (colored by COG categories), Genes on reverse strand (colored by COG categories), RNA genes (tRNAs green, rRNAs red, other RNAs black), GC content(black), GC skew (purple/olive).

The genome consist of one circular chromosome of 2,039,943 bp length with a 40.3% G+C content (Table 3 and Figure 3). Of the 2,066 genes predicted, 2,015 were protein-coding genes, and 51 RNAs; 69 pseudogenes were also identified. The majority of the protein-coding genes (74.3%) were assigned a putative function while the remaining ones were annotated as hypothetical proteins. The distribution of genes into COGs functional categories is presented in Table 4.

**Table 3:** Genome Statistics

Attribute	Value	% of Total
Genome size (bp)	2,039,943	100.00%
DNA coding region (bp)	1,859,937	91.18%
DNA G+C content (bp)	822,588	40.32%
Number of replicons	1	
Extrachromosomal elements	0	
Total genes	2,066	100.00%
RNA genes	51	2.47%
rRNA operons	1	
tRNA genes	46	2.23%
Protein-coding genes	2,015	97.53%
Pseudo genes	69	3.34%
Genes with function prediction (proteins)	1,535	74.30%
Genes in paralog clusters	912	44.14%
Genes assigned to COGs	1,719	83.20%
Genes assigned Pfam domains	1,704	82.48%
Genes with signal peptides	327	15.83%
Genes with transmembrane helices	549	26.57%
CRISPR repeats	7	

**Table 4:** Number of genes associated with the general COG functional categories

Code	value	%age	Description
J	138	7.2	Translation, ribosomal structure and biogenesis
A	0	0.0	RNA processing and modification
K	85	4.5	Transcription
L	108	5.7	Replication, recombination and repair
B	2	0.1	Chromatin structure and dynamics
D	22	1.2	Cell cycle control, cell division, chromosome partitioning
Y	0	0.0	Nuclear structure
V	26	1.4	Defense mechanisms
T	79	4.1	Signal transduction mechanisms
M	79	4.1	Cell wall/membrane biogenesis
N	68	3.6	Cell motility
Z	0	0.0	Cytoskeleton
W	0	0.0	Extracellular structures
U	43	2.3	Intracellular trafficking and secretion, and vesicular transport
O	58	3.0	Posttranslational modification, protein turnover, chaperones
C	128	6.7	Energy production and conversion
G	211	11.0	Carbohydrate transport and metabolism
E	201	10.5	Amino acid transport and metabolism
F	60	3.1	Nucleotide transport and metabolism
H	77	4.0	Coenzyme transport and metabolism
I	35	1.8	Lipid transport and metabolism
P	99	5.2	Inorganic ion transport and metabolism
Q	20	1.1	Secondary metabolites biosynthesis, transport and catabolism
R	238	12.5	General function prediction only
S	134	7.0	Function unknown
-	347	16.8	Not in COGs

## Insights into the genome sequence

**Figure 4:**
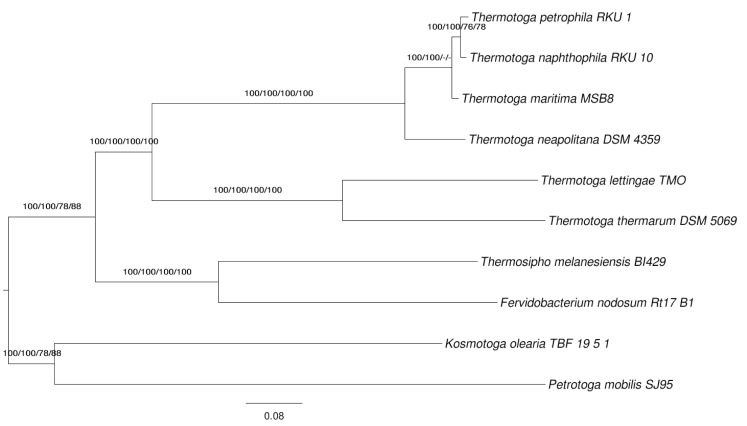
Phylogenetic tree inferred from completely sequenced genomes of the ‘*Thermotogae*’ type strains. The tree was inferred from 360,527 aligned amino acid characters under the maximum likelihood (ML) criterion and rooted using midpoint rooting [11]. The branches are scaled in terms of the expected number of substitutions per site. Numbers above the branches are bootstrapping support values (if larger than 60%) from (i) maximum-likelihood supermatrix analysis; (ii) maximum-parsimony supermatrix analysis; (iii) maximum-likelihood gene-content analysis; (iv) maximum-parsimony gene-content analysis.

Because a number of complete genome sequences of type strains from the phylum has already been published, we conducted a phylogenomic analysis using the bioinformatics pipeline established in [48] and further modified as described in [18,49]. The resulting supermatrix comprised 1,889 genes and 582,906 characters before, 1,168 genes and 360,527 characters after cleaning with MARE. The selected model was PROTGAMMALGF; the resulting tree had a log likelihood of -3,783,776.37 and is shown in Figure 3. The best maximum-parsimony tree found had a length of 404,859 steps (not counting uninformative characters) and was topologically identical. The gene-content matrix comprised 3,267 characters and yielded best trees with a log likelihood of -13,904.74 and a parsimony score of 2,243, respectively. Bootstrapping support values from all four applied methods are shown in Figure 4 if larger then 60%.The phylogenomic trees disagree with the 16S rRNA tree (Fig. 1) in some respects. For instance, *Thermosipho* appears as a sister group of *Fervidobacterium*. Hence we assessed whether the 16S rRNA alignment described above, if reduced to the strains used in the phylogenomic analysis, is in significant conflict with the phylogenomic topology. Using the kind of constraint analysis mentioned above, search under the maximum-likelihood criterion yielded a best tree with a score of -5,425.82 if the search was unconstrained but a tree with a log likelihood of -5,436.37 under the constraint; this was not significantly worse in the SH test as implemented in RAxML (α = 0.05). Under maximum parsimony, the globally best trees had a score of 512, whereas the best constrained tree was 529 steps in length; this was significantly worse according to KH test implemented in PAUP* (p = 0.0148).

Currently there is neither evidence for a significant discrepancy between 16S rRNA and whole-genome data, nor a significant disagreement between 16S rRNA and the classification after *Thermococcoides shengliensis* was placed in *Kosmotoga* as *K. shengliensis* (see above). Nevertheless, as usual [36] the phylogenomic trees are much better resolved than the 16S rRNA phylogenies, and the *Kosmotoga-Thermococcoides* question could also be addressed in greater detail if the genomes of the type strains were available, as digital replacements of DNA-DNA hybridization have been implemented [23]. The classification of the group thus can only benefit from additional genome-sequenced type strains.

The *T. thermarum* genome has numerous differences from the other *Thermotoga* genomes, particularly with regard to cofactor metabolism. Some of these differences are shared with *T. lettingae*, which is more closely related to *T. thermarum* than the other *Thermotoga* species with sequenced genomes (Figs. 1 and 4). There appears to be a significant difference in Fe-S cluster synthesis between *T. thermarum* and the other *Thermotoga* species. Fe-S cluster synthesis requires at the minimum a cysteine desulfurase to produce sulfur and a scaffold protein for Fe-S cluster assembly (reviewed in [50]). There are three Fe-S cluster biosynthesis pathways in bacteria: Nif, Isc, and Suf [51]. *T. maritima* uses the Suf system. It has an operon with sufCBDSU genes and another operon with a second copy of sufCB [51]. The SufS protein is a cysteine desulfurase. In *Bacillus subtilis*, which has a similar set of Suf proteins as *T. maritima*, the SufU protein has been shown to be a scaffold protein [52]. In *Escherichia coli*, which lacks the SufU protein, SufB is a scaffold protein, and SufC and SufD are required for iron acquisition [53]. In E. coli the Suf genes are expressed under iron starvation conditions [51]. *T. maritima*, therefore, may have two scaffold proteins, SufU and SufB. *T. thermarum* has a cluster of four genes (Theth_0902-0905) including two cysteine desulfurases and two proteins similar to SufU, but the SufBCD proteins are not present in the *T. thermarum* genome. Thus *T. thermarum* appears to encode a minimal set of Fe-S cluster synthesis proteins. It is possible that in *Thermotogales* and *Firmicutes* SufU is used as the scaffold protein if iron is plentiful, while SufBCD is required under low-iron conditions. *T. thermarum* may have access to more iron in its environment than other *Thermotoga* species. Interestingly, adjacent to the Fe-S cluster biosynthesis genes in *T. thermarum* is a transporter for which the closest characterized homolog is ZupT from *E. coli*, which transports iron and other divalent metals [54]. *T. lettingae* has similar Fe-S cluster synthesis genes as *T. thermarum* but also encodes the sufCB genes.

All of the *Thermotoga* species lack uroporphyrinogen synthesis and most of vitamin B12 synthesis, and the only enzyme of B12 metabolism common to all *Thermotoga* genomes is the adenosyltransferase that produces adenosylcobalamin from cobalamin. However, *T. thermarum* contains several genes clustered together (Theth_1729-1737) involved in the later steps of cobalamin synthesis, suggesting that it can utilize precursors of cobalamin that the other *Thermotoga* species can not utilize. Most of these genes are also found in T. lettingae. *T. thermarum* and T. lettingae are the only *Thermotoga* species to have genes for tungsten-dependent aldehyde:ferredoxin oxidoreductases (Theth_0853, Theth_1019). Theth_0853 has 68% amino acid identity to the formaldehyde:ferredoxin oxidoreductase of Pyrococcus furiosus, suggesting it was recently acquired. These enzymes use a bis-molybdopterin form of molybdenum cofactor with tungsten in place of molybdenum [55]. *T. thermarum* and *T. lettingae* are also the only *Thermotoga* species to have genes for tungsten transport (Theth_0538-540) and molybdopterin biosynthesis (Theth_0439-440, Theth_0535-536, Theth_1749). However, genes for molybdopterin synthase (moaD, moaE) could not be identified, suggesting they may have alternative genes for this step of the pathway. *T. thermarum* also has molybdenum cofactor guanylyltransferase (Theth_0112) for production of molybdopterin guanine dinucleotide. Adjacent to this enzyme are a formate dehydrogenase accessory protein, a formate transporter pseudogene, and a molybdopterin dinucleotide-dependent formate dehydrogenase pseudogene. There are no other genes in *T. thermarum* with the molybdopterin dinucleotide binding domain (pfam01568) suggesting that molybdopterin dinucleotide synthesis is no longer necessary.

*T. thermarum* has fewer glycosyl hydrolases than the other *Thermotoga* species [56], but it has genes for transport and utilization of oligogalacturonides that are not present in the others. *T. thermarum* has an ABC transporter (Theth_0394-0396) similar to the oligogalacturonide ABC transporter from Erwinia chrysanthemi [57], while none of the other *Thermotoga* genomes contains genes similar to any of the known oligogalacturonide transporters. Close to the transporter is the kduI gene (Theth_0398) involved in oligogalacturonide degradation, which is also not found in other *Thermotoga* species. The transporter genes and kduI gene have 60-70% amino acid identity to genes from *Thermoanaerobacter*, suggesting recent acquisition from Clostridia. Other genes found only in *T. thermarum* and *T. lettingae* include enzymes for histidine degradation (Theth_0380, Theth_1683, Theth_0980) and serine degradation (Theth_1895-1896). *Thermotoga* species generally grow on a variety of carbohydrates, but the presence of these pathways suggests amino acids may be a carbon and energy source for some species.All *Thermotoga* species have genes for the Rnf complex, which couples an ion gradient to the transfer of electrons between NADH and ferredoxin. In addition *T. thermarum* and T. lettingae have genes for the NqrBCDEF subunits of a sodium-translocating NADH:quinone dehydrogenase (Theth_1137-1141). They lack the NqrA subunit, which contains the quinone binding site [58], so the other participant in the reaction (besides NADH) is unknown.
